# Imaging biomarkers of cortical neurodegeneration underlying cognitive impairment in Parkinson’s disease

**DOI:** 10.1007/s00259-025-07070-z

**Published:** 2025-01-31

**Authors:** Jesús Silva-Rodríguez, Miguel Ángel Labrador-Espinosa, Sandra Castro-Labrador, Laura Muñoz-Delgado, Pablo Franco-Rosado, Ana María Castellano-Guerrero, Daniel Macías-García, Silvia Jesús, Astrid D. Adarmes-Gómez, Fátima Carrillo, Juan Francisco Martín-Rodríguez, David García-Solís, Florinda Roldán-Lora, Pablo Mir, Michel J. Grothe

**Affiliations:** 1 Reina Sofia Alzheimer Center, CIEN Foundation, ISCIII, Madrid, Spain; 2 Unidad de Trastornos del Movimiento, Servicio de Neurología, Instituto de Biomedicina de Sevilla, Hospital Universitario Virgen del Rocío/CSIC/Universidad de Sevilla, Sevilla, Spain; 3 Centro de Investigación Biomédica en Red sobre Enfermedades Neurodegenerativas (CIBERNED), ISCIII, Madrid, Spain; 4 Department of Psychiatry and Neurochemistry, Institute of Physiology and Neuroscience, University of Gothenburg, Gothenburg, Sweden; 5 Departamento de Psicología Experimental, Facultad de Psicología, Universidad de Sevilla, Sevilla, Spain; 6 Servicio de Medicina Nuclear, Hospital Universitario Virgen del Rocío, Sevilla, Spain; 7 Unidad de Radiodiagnóstico, Hospital Universitario Virgen del Rocío, Sevilla, Spain; 8 Fundación CIEN, Centro Alzheimer Reina Sofía, C. de Valderrebollo, 5, Vallecas, Madrid, 28031 Spain; 9 Unidad de Trastornos del Movimiento, Instituto de Biomedicina de Sevilla, Hospital Universitario Virgen del Rocío, Avda. Manuel Siurot s/n, Seville, 41013 Spain

**Keywords:** Parkinson’s disease, Cognitive decline, [^18^F]FDG PET, MRI, DTI, Hypometabolism, Atrophy, Mean diffusivity

## Abstract

**Purpose:**

Imaging biomarkers bear great promise for improving the diagnosis and prognosis of cognitive impairment in Parkinson’s disease (PD). We compared the ability of three commonly used neuroimaging modalities to detect cortical changes in PD patients with mild cognitive impairment (PD-MCI) and dementia (PDD).

**Methods:**

53 cognitively normal PD patients (PD-CN), 32 PD-MCI, and 35 PDD underwent concurrent structural MRI (sMRI), diffusion-weighted MRI (dMRI), and [^18^F]FDG PET. We extracted grey matter volumes (sMRI), mean diffusivity (MD, dMRI), and standardized uptake value ratios ([^18^F]FDG PET) for 52 cortical regions included in a neuroanatomical atlas. We assessed group differences using ANCOVA models and further applied a cross-validated machine learning approach to identify the modality-specific brain regions that are most indicative of dementia status and assessed their diagnostic accuracy for group separation using receiver operating characteristic analyses.

**Results:**

In sMRI, atrophy of temporal and posterior-parietal areas allowed separating PDD from PD-CN (AUC = 0.77 ± 0.07), but diagnostic accuracy was poor for separating PD-MCI from PD-CN (0.57 ± 0.10). dMRI showed most pronounced diffusivity changes in the medial temporal lobe, which provided excellent diagnostic performance for PDD (AUC = 0.87 ± 0.06), and a more modest but still significant performance for PD-MCI (AUC = 0.71 ± 0.09). Finally, [^18^F]FDG PET revealed pronounced hypometabolism in posterior-occipital regions, which provided the highest diagnostic accuracies for both PDD (AUC = 0.89 ± 0.05) and PD-MCI (AUC = 0.78 ± 0.05). In statistical comparisons, both [^18^F]FDG PET (*p* < 0.001) and dMRI (*p* < 0.031) outperformed sMRI for detecting PDD and PD-MCI.

**Conclusion:**

Among the tested modalities, [^18^F]FDG PET was most accurate for detecting cortical changes associated with cognitive impairment in PD, especially at early stages. Diffusion measurements may represent a promising MRI-based alternative.

**Supplementary Information:**

The online version contains supplementary material available at 10.1007/s00259-025-07070-z.

## Introduction

Cognitive impairment (CI) is one of the most common and devastating non-motor symptoms of Parkinson’s disease (PD) [1]. At disease onset, symptoms of mild cognitive impairment (MCI) are already present in approximately 20% of PD patients (PD-MCI), and long-term epidemiological studies have shown that the large majority (~ 80%) of PD patients will eventually develop a PD-associated dementia syndrome (PDD) [2, 3]. The underlying mechanisms of cognitive decline and dementia associated with PD remain unclear. Cortical involvement of Lewy body pathology is considered a key feature, but multiple mechanisms are likely involved [4]. Thus, the identification of biomarkers that may identify brain changes associated with PD-related CI could provide important insights into the involved pathological processes and improve accurate diagnosis [5].

In recent years, there has been growing interest in using neuroimaging to detect early cortical changes associated with PD-CI [6, 7]. Structural magnetic resonance imaging (sMRI) has been extensively employed to measure grey matter atrophy in PD-CI, yet the findings have been inconsistent across studies [7, 8]. While some studies have identified atrophy in various cognition-relevant areas [9–11], others have failed to replicate these findings [12, 13]. Moreover, the specific cortical areas reported to exhibit atrophy have varied widely across different studies [7]. By contrast, [^18^F]fluorodeoxyglucose ([^18^F]FDG) positron emission tomography (PET) has revealed a more consistent pattern of posterior-occipital brain hypometabolism associated with PD-CI, which can already be observed in PD-MCI patients several years before progression to dementia [14, 15].

While direct comparisons between [^18^F]FDG PET and MRI are rare, a recent meta-analysis across uni-modal PET and MRI imaging studies concluded that hypometabolism on [^18^F]FDG PET is a more sensitive and consistent imaging marker of the neurodegenerative changes in PD [16]. Similarly, one previous multimodal imaging study directly comparing the performance of sMRI and [^18^F]FDG PET in PD-CI concluded that changes in hypometabolism precede and exceed atrophy in the course of the disease [17], especially in posterior-occipital regions. More recently, tissue diffusion indices derived from diffusion-weighted MRI (dMRI) have shown promise in detecting early microstructural alterations that occur before apparent atrophy on sMRI in the course of PD [13, 18, 19]. Considering the comparably limited availability of [^18^F]FDG PET, dMRI may be an attractive, MRI-based alternative for assessing PD-CI in the clinic. However, multimodal imaging studies allowing a direct comparison between [^18^F]FDG PET and dMRI are still lacking.

The aim of the current study was to provide a direct comparison of the sensitivity and diagnostic utility of sMRI, dMRI, and [^18^F]FDG PET as neuroimaging biomarkers of PD-CI, using a relatively large cohort of well-phenotyped PD patients spanning the clinical continuum from cognitive normality (PD-CN) over PD-MCI to PDD. Additionally, we also evaluated the potential of combining multimodal imaging information to improve the detection of pathological changes associated with CI in PD.

## Methods

### Participants

Our study sample included 120 patients with PD prospectively recruited by the Movement Disorders Unit at the University hospital ‘Virgen del Rocío’ in Seville, Spain. PD diagnosis was performed following the Movement Disorder Society (MDS) Clinical Diagnostic Criteria [20], and cognitive performance was evaluated using the Parkinson’s Disease Cognitive Rating Scale (PD-CRS). The sample was intentionally enriched for PD-CI during recruitment, and patients were diagnosed as having PD with normal cognition (PD-CN, PD-CRS > 81; *N* = 53), PD-MCI (81 ≥ PD-CRS > 64; *N* = 32) [21], or PDD (PD-CRS ≤ 64 + confirmed functional impairment; *N* = 35) [22]. The Montreal Cognitive Assessment (MoCA) scale was used as a complementary measurement of global cognitive performance. Cognitive assessment was performed while patients were in the ON state. Patients on cholinergic treatment were not excluded, but complementary analyses excluding this group are presented. Motor status was assessed using Part III of the Unified Parkinson’s Disease Rating Scale (UPDRS-III) and the Modified Hoehn and Yahr scale (H&Y), both conducted while the patients were in the OFF state, defined as a period of at least 12 h without dopaminergic medication. The study protocol was approved by the Ethics Committee of the University hospital ‘Virgen del Rocío’ (Date: 08/01/2021, approval number: 2158-N-20) according to the guidelines of the Helsinki declaration, and written informed consent was obtained from all study participants.

### Image acquisition

All patients underwent a multimodal MRI acquisition in a Philips Ingenia 3T MRI scanner. sMRI images were acquired using a high-resolution 3D T1-weighted (T1W 3D TFE SENSE) sequence (TR = 8.2 ms, TE = 3.75 ms, flip angle = 8 µ, acquisition matrix = 256 × 256 × 180, voxel size = 0.94 × 0.94 × 1 mm^3^), while dMRI data was acquired using a diffusion-weighted spin-echo echo-planar-imaging (DWI-EPI) pulse sequence with 32 non-collinear diffusion gradient orientations (b = 1000 s/mm2) in addition to a non-weighted (B0) image. Resulting dMRI images had a field of view (FOV) of 224 × 224 mm^2^ over a 128 × 128 matrix with 60 slices. Slice thickness was 2 mm, with no gaps.

[^18^F]FDG PET acquisitions were performed at a separate visit in temporal proximity to the MRI scan (Δt = 0.3 ± 0.5 years). Patients were scanned for 20 min, 45 min after the injection of ∼200 Mbq of [^18^F]FDG. Due to a hardware upgrade during the recruitment phase, acquisitions were performed on two different scanners, a Siemens BioGraph HiRes (19 patients: 9 PD-CN, 5 PD-MCI, 5 PDD) and a GE Discovery MI (101 patients: 44 PD-CN, 27 PD-MCI, 30 PDD). Reconstruction was performed using the 3D iterative reconstruction methods implemented in each scanner (*Siemens*: FORE + OSEM-2D; *GE*: VPHD), including corrections for attenuation, scatter, and random coincidences.

### Image processing and analysis

sMRI images were segmented into grey matter (GM), white matter (WM), and cerebrospinal fluid (CSF) partitions, and spatially normalized to Montreal Neurological Institute (MNI) space using the standardized routines provided by the Computational Anatomy Toolbox (CAT12) of the Statistical Parametric Mapping software (SPM12, https://www.fil.ion.ucl.ac.uk/spm/). [^18^F]FDG PET images were normalized to MNI space using SPM12 and smoothed to an isotropic 8-mm resolution by applying differential smoothing values for each scanner (Siemens: 4.5 mm, GE: 6.5 mm) [23], which were calculated using a previously validated resolution estimation method aimed at calculating effective image resolution directly from brain images instead of using dedicated phantoms [24]. Finally, a previously validated data-driven histogram-based intensity normalization algorithm was applied [25, 26].

dMRI volumes were first processed with FSL (https://www.fmrib.ox.ac.uk/fsl) to correct for head motion and eddy-current distortions, and to remove voxels outside of the brain. Registration-based distortion correction was performed by applying diffeomorphic transformations between the dMRI images and sMRI using the Advanced Normalization Tools (ANTs, https://github.com/ANTsX/ANTs) [27]. After pre-processing, images were reconstructed using the computational routines provided by Pasternak et al. [28] for obtaining free water-corrected mean diffusivity (MD) maps [19]. MD has been reported to be a sensitive diffusion index of microstructural cortical changes that may precede volumetric changes [29], and free-water correction may play an important role in evaluating cortical diffusivity, as it helps to remove CSF contamination, a particular type of partial volume effect that occurs along the contour lines of the ventricles and around the perimeters of the brain parenchyma in voxels shared by CSF and brain tissue [28]. Taking advantage of this processing, we also derived free water-corrected fractional anisotropy (FA) values and the fraction of free water in the brain’s extracellular space (FW) as complementary dMRI metrics [30]. MD, FA, and FW maps were transformed to MNI space using the transformations of the co-registered sMRI. Normalized images were then masked using the segmented GM derived from CAT12.

After preprocessing, for each modality quantitative values were extracted within 52 cortical regions-of-interest (ROI) as defined in the Harvard-Oxford neuroanatomical atlas. For sMRI, the GM volume for each ROI was calculated by summing up the modulated GM voxel values within the ROI. Values were normalized by the total intracranial volume (TIV), calculated as the total sum of GM, WM, and CSF partitions. Mean [^18^F]FDG PET standardized uptake value ratios (SUVR) were calculated as the average signal of the intensity normalized PET image across voxels within each ROI. Similarly, average MD was obtained as the mean value within each of the atlas-defined ROIs.

### Statistical analysis

Demographic, clinical, and biomarker variables were compared using two-sample t-tests for normally distributed continuous variables and Fisher’s exact tests for categorical variables.

Brain-wide group-level analysis was performed using an ROI-based approach that allowed an objective comparison of the regional effects between modalities. Independent ANCOVA comparisons (PD-CN vs. PD-MCI, PD-CN vs. PDD) were performed for each of the ROIs using the previously calculated GM, MD, and SUVR values. Age and motor symptom severity (UPDRS-III) were used as covariates to isolate changes related to cognition. Results are reported as effect size (Cohen’s d) at an FDR-corrected threshold of *p* < 0.05. For convenience, Cohen’s d values were defined to be positive in the direction of increased neurodegeneration for each modality (i.e., decreased GM, decreased SUVR, increased MD). In addition to the group comparisons, we also performed complementary partial Pearson correlation analysis between the PD-CRS and regional GM, MD, and SUVR values (also using age and UPDRS-III as covariates).

To assess the diagnostic performance of each neuroimaging modality in a clinical scenario, we followed a machine learning approach based on a previously developed framework for comparing regional neuroimaging biomarkers from multiple modalities [31]. Supplementary Fig. 1 shows a schematic of the used methodology. Briefly, we iteratively trained a penalized logistic regression model with an elastic net penalty and tested its diagnostic performance across independent training-test splits of the study sample (1000 iterations). First, the PDD and PD-CN data were divided at each iteration into 2/3 − 1/3 train-test splits, and the train sets were used to estimate the penalized regression model. Penalization shrinks the less contributive regression coefficients by imposing a penalty on the size of the correlation strength, which can result in the exclusion of the least informative variables from the regression model by shrinking their respective regression coefficients to zero [31]. Preserved features with non-zero coefficients may thus be considered as those that are relevant to the classification problem for the given train-test split. In order to avoid over-fitting and data-leakage, for each iteration the hold out test set was then used for an unbiased evaluation of the performance of the model for distinguishing between PD-CN and PDD as well as for distinguishing between PD-CN and PD-MCI (PD-CN test set vs. whole PD-MCI sample). For each train-test iteration of the PDD and PD-MCI classification tasks, we calculated the relative contribution of each ROI to the given model (model coefficients normalized to percentage contribution), the receiver operating characteristics (ROC) curves and corresponding area under the curve (AUC) values, as well as the maximum Youden Indices (Max(J)) and corresponding sensitivities, specificities, and accuracies. These variables were then averaged across the 1000 iterations to obtain robust and unbiased estimates of the ROI contributions and performance metrics for each classification task.

Of note, in our main analysis we restricted the training phase to the PD-CN vs. PDD classification task to ensure that we identified those neuroimaging features that are robustly linked to dementia, and we then tested whether these abnormalities can already be detected and used to classify earlier, predementia stages of CI in PD (i.e., PD-MCI). The rationale of this approach lies in its potential clinical utility for the specific detection of dementia-related imaging abnormalities in PD-MCI [15]. However, PD-MCI is a heterogenous entity, including neurodegeneration phenotypes that may not be directly related to dementia [32], and focusing on dementia-related imaging features may potentially miss relevant information for the identification of MCI in PD. Thus, in complementary analyses we trained and tested equivalent models directly aimed at distinguishing between PD-MCI and PD-CN.

In addition to the modality-specific models described above, the same methodology was applied to test the added value of a fully multi-modal model including features from [^18^F]FDG PET, sMRI, and dMRI, as well as of a separate model combining only features from sMRI and dMRI. The latter holds particular interest as both sequences can be acquired in the same MRI imaging session.

Statistical significance of differences between AUC values of the different classifiers models was assessed using DeLong tests [33]. All models were implemented using the scikit-learn library v.1.3.0 running on Python 3.10 (www.scikit-learn.org) and included age and motor symptom severity (UPDRS-III) as covariates. All the main analyses were repeated in sensitivity analyses using subsamples excluding patients that (a) were under cholinergic treatment at the start of the study, or (b) were acquired using an older scanner model (Siemens BioGraph HiRes). Furthermore, we have also conducted complementary analyses exploring the brain-wide group-level differences in GM, SUVR, and MD between PD-MCI and PDD patients, as well as the performance of modality-specific classifiers for this task that were trained using the procedure described above.

## Results

### Demographics

Table 1 summarizes demographical and clinical data of our cohort. While there were no differences in sex between groups, both PD-MCI and PDD patients were significantly older than PD-CN (*p* < 0.004) and also had an older age of disease onset (*p* < 0.02). Disease duration was longer for PDD patients compared to both PD-CN (d = 1.09, *p* < 0.001) and PD-MCI (d = 0.81, *p* = 0.002), but was similar between PD-CN and PD-MCI. Nine patients were on rivastigmine treatment, all of them pertaining to the PDD group (9/35). Regarding motor symptoms (UPDRS-III), both CI groups were significantly more impaired than PD-CN (PDD: d = 1.29, *p* < 0.001; PD-MCI: d = 0.64, *p* = 0.007).

### ROI analysis

**Fig. 1:**
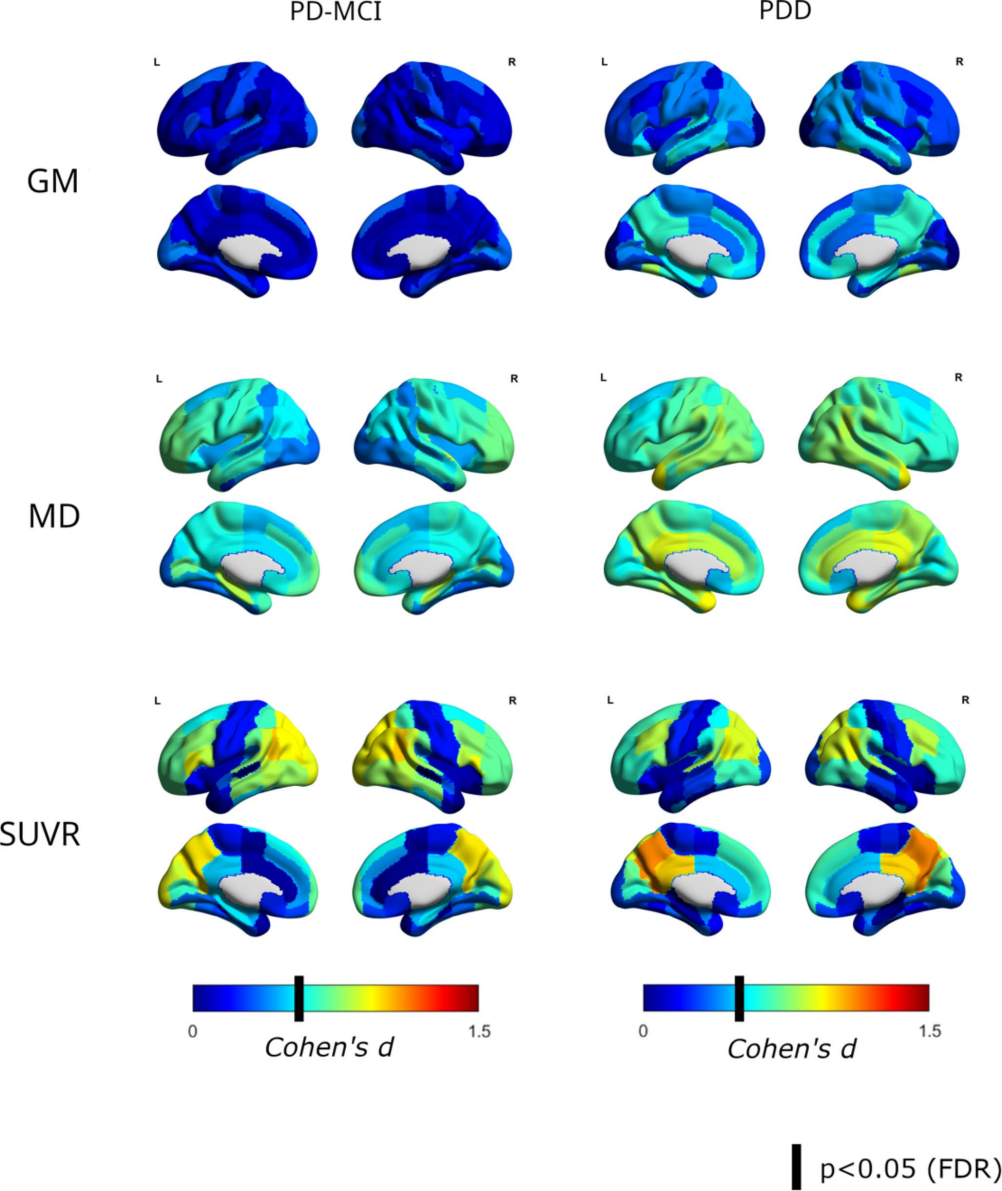
ROI-based analysis results for the different modalities (from top to bottom), for PD-MCI (left) and PDD (right). Color scale represents effect size (Cohen’s d), and the solid bar in the color scale represents the threshold for p(FDR) < 0.05

Brain-wide ROI-based analysis results are visualized in Fig. 1 and detailed statistics are reported in Supplementary Tables 1, 2, and 3. In sMRI, PDD patients showed significant atrophy compared to PD-CN in posterior-parietal (notably the precuneus and the posterior cingulate) and lateral and medial temporal areas. The most affected ROI was the temporo-occipital fusiform cortex (d = 0.80, *p* = 0.03). However, only very mild changes were observed in patients with PD-MCI compared to PD-CN, where no region reached the threshold for statistical significance. In dMRI, PDD patients showed a significant brain-wide increase in MD, which was most notable in the temporal pole (d = 0.93), posterior cingulate (d = 0.90), and angular gyrus (d = 0.83). PD-MCI patients also showed significant alterations in MD, which were most notable in the hippocampus (d = 0.83). Qualitatively, dMRI provided larger effect sizes and more extended effects compared to sMRI, especially for PD-MCI patients. Complementary diffusion metrics, including FA and FW, were also assessed but showed generally smaller effect sizes than MD for measuring cortical changes (Supplementary Fig. 2). Finally, [^18^F]FDG PET revealed significant hypometabolism in posterior-occipital regions in PDD, especially notable in the precuneus (d = 1.11), posterior cingulate (d = 1.00), and angular gyrus (d = 0.92). In contrast to dMRI and especially sMRI, these changes were also pronounced in PD-MCI patients with a very similar pattern, especially affecting the angular gyrus (d = 1.02) and precuneus (d = 0.97). The reverse contrasts (i.e., more neurodegeneration in PD-CN vs. PD-MCI or PDD) did not show any significant effect for any of the modalities. Findings remained largely unchanged when excluding the 9 PDD patients on cholinergic treatment (Supplementary Fig. 3), or the 19 patients scanned on the Siemens BioGraph HiRes (Supplementary Fig. 4).

We also performed complementary brain-wide ROI-based analyses comparing the PD-MCI and PDD groups directly. Differences were spatially similar to those of the PDD vs. PD-CN comparison, but with lower regional effect sizes (Supplementary Fig. 5A). Finally, complementary partial Pearson correlation analysis between PD-CRS scores and the different regional imaging features revealed similar results as the categorical comparison between diagnostic groups, with similar modality-specific regional patterns and markedly higher effect sizes (partial Pearson’s r) for [^18^F]FDG PET compared to the MRI modalities. (Supplementary Fig. 6).

**Table 1:** Demographical and clinical data for the different study groups

	PD-CN (*n* = 53)	PD-MCI (*n* = 32)	PDD (*n* = 35)	PD-CN vs. PD-MCI	PDD vs. PD-CN	PDD vs. PD-MCI
Age, y	60.5 ± 8.4	65.8 ± 7.3	71.7 ± 7.2	d = 0.66 *p* = 0.004^(*)^	d = 1.41 *p* < 0.001^(*)^	d = 0.81 *p* = 0.002^(*)^
Female, %	22.6	18.9	25.7	*p* = 0.787	*p* = 0.801	*p* = 0.566
PD onset age, y	55.1 ± 8.9	59.8 ± 8.2	61.0 ± 9.7	d = 0.53 *p* = 0.020^(*)^	d = 0.63 *p* = 0.004^(*)^	d = 0.14 *p* = 0.580
Disease Duration, y	5.3 ± 3.3	6.0 ± 4.6	10.7 ± 6.5	d = 0.17 *p* = 0.44	d = 1.09 *p* < 0.001^(*)^	d = 0.81 *p* = 0.002^(*)^
UPDRS-III	17.6 ± 8.8	23.1 ± 8.7	33.4 ± 16.1	d = 0.63 *p* = 0.007^(*)^	d = 1.29 *p* < 0.001^(*)^	d = 0.78 *p* = 0.002^(*)^
H&Y	1.9 ± 0.5	2.2 ± 0.5	2.5 ± 0.8	d = 0.59 *p* = 0.01^(*)^	d = 0.93 *p* < 0.001^(*)^	d = 0.43 *p* = 0.08
MoCA, score	25.8 ± 2.6	21.7 ± 3.4	13.9 ± 4.2	d=-1.40 *p* < 0.001^(*)^	d=-3.56 *p* < 0.001^(*)^	d=-2.01 *p* < 0.001^(*)^
PD-CRS, total score	100.0 ± 10.7	74.8 ± 3.9	46.8 ± 12.1	d=-2.83 *p* < 0.001^(*)^	d=-4.64 *p* < 0.001^(*)^	d=-3.00 *p* < 0.001^(*)^
PD-CRS, fronto- subcortical score	70.7 ± 10.4	47.3 ± 4.3	24.1 ± 9.8	d=-2.69 *p* < 0.001^(*)^	d=-4.54 *p* < 0.001^(*)^	d=-2.96 *p* < 0.001^(*)^
PD-CRS, posterior- cortical score	29.2 ± 1.0	27.5 ± 2.1	22.7 ± 4.1	d=-1.14 *p* < 0.001^(*)^	d=-2.39 *p* < 0.001^(*)^	d=-1.43 *p* < 0.001^(*)^

^(*)^ significantly different with *p* < 0.05. Abbreviations: PD, Parkinson’s Disease; CN, cognitively normal; MCI, mild cognitive impairment; PDD, Parkinson’s Disease Dementia; UPDRS-III, Unified Parkinson’s Disease Rating Scale, part III (motor section); H&Y, Hoehn & Yahr scale; MoCA, Montreal Cognitive Assessment scale; PD-CRS, Parkinson’s Disease Cognitive Rating Scale

### Modality-specific classification models

**Fig. 2:**
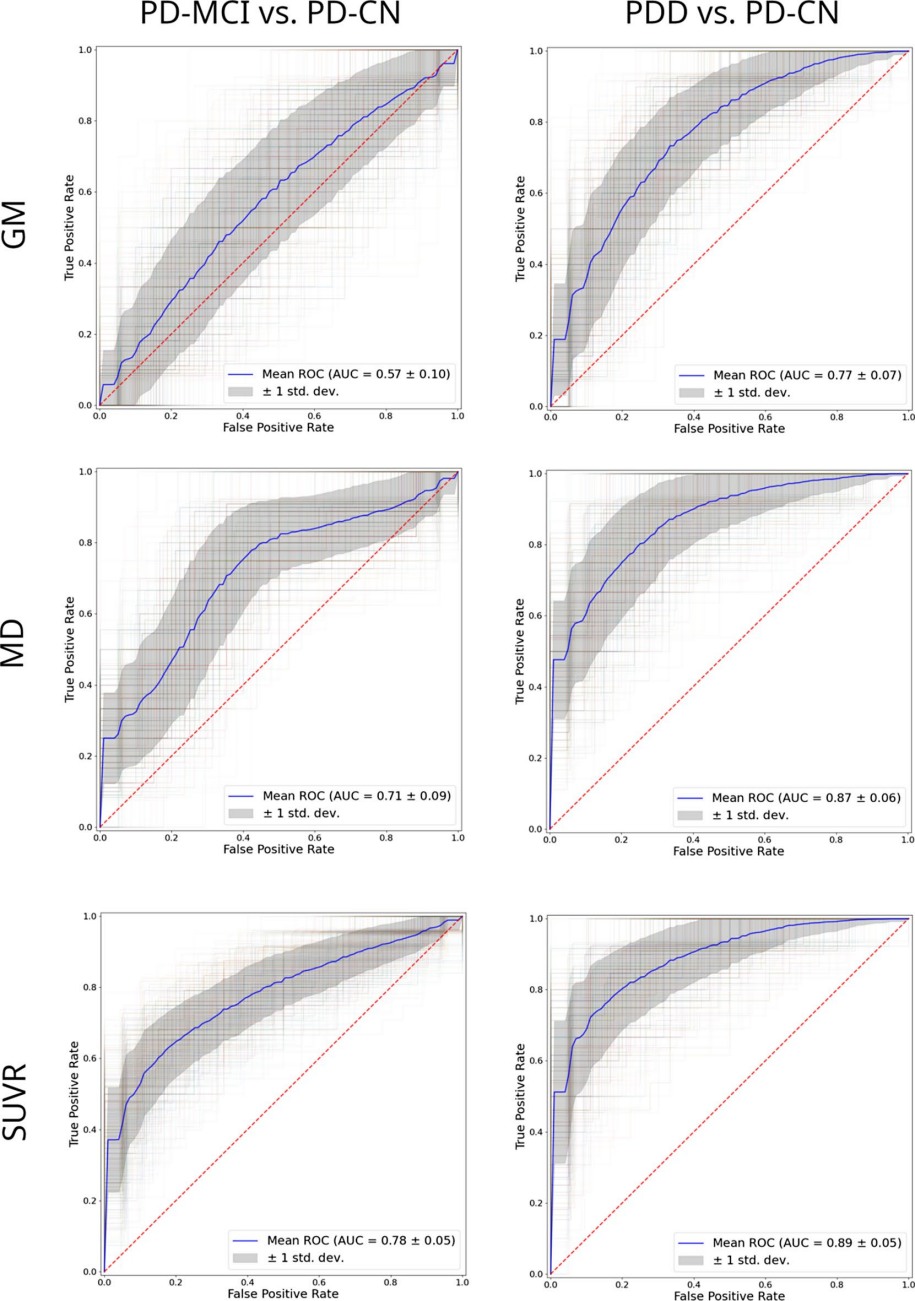
Receiver Operating Characteristic (ROC) curves for the different modality-specific models (blue). Grey areas present the standard deviation of the averaged ROC curves

Figure 2 shows the ROC curves of the classifiers for each modality, and Table 2 provides a more complete description of the classification performance of the modality-specific classifiers. sMRI showed a reasonable performance for classifying between PD-CN and PDD (AUC = 0.77 ± 0.07, Max(J) = 0.51±0.11), where the temporooccipital fusiform gyrus (18.1% average contribution), the amygdala (18.1%), the anterior part of the middle temporal gyrus (12.4%), and the precuneus (11.3%) were the ROIs that most contributed to the models (Supplementary Fig. 7, top). However, the sMRI-based models only provided chance-level performance for the discrimination between PD-CN and PD-MCI (AUC = 0.57 ± 0.10, Max(J) = 0.38±0.13). Regarding dMRI, MD of the planum polare (38.4% average contribution), the hippocampus (19.1%), and Heschl’s gyrus (19.1%) showed the highest contributions for discriminating between PD-CN and PDD (Supplementary Fig. 7, Center). In the independent test data, the resulting models provided an excellent classification performance for PDD vs. PD-CN (AUC = 0.87 ± 0.06, Max(J) = 0.67±0.10), and a more modest but still significant discrimination between PD-MCI and PD-CN (AUC = 0.71 ± 0.09, Max(J) = 0.47±0.12). DeLong tests showed that dMRI outperformed sMRI for the discrimination of both PDD (*p* = 0.031) and PD-MCI (*p* = 0.009). Finally, for [^18^F]FDG PET, the most prominent features for distinguishing between PD-CN and PDD were the precuneus (51.4% average contribution) and the superior part of the lateral occipital cortex (19.4%) (Supplementary Fig. 7, bottom). In the test data, the [^18^F]FDG PET-based classifiers yielded an excellent performance for discriminating between PD-CN and PDD (AUC = 0.89 ± 0.05, Max(J) = 0.75±0.10), and a notable performance for discriminating between PD-CN and PD-MCI (AUC = 0.78 ± 0.05, Max(J) = 0.53±0.11). In DeLong tests, [^18^F]FDG PET significantly outperformed sMRI (*p* < 0.001) for the classification of PDD, and both sMRI (*p* < 0.001) and dMRI (*p* = 0.03) for the classification of PD-MCI.

Classification experiments for the PD-CN vs. PDD task were replicated excluding 9 PDD patients that were on cholinergic treatment, providing almost identical results: GM, AUC = 0.75 vs. 0.77 using the full PDD sample; MD, AUC = 0.90 vs. 0.87; [^18^F]FDG PET, AUC = 0.85 vs. 0.89. Similarly, exclusion of the 19 patients scanned on the Siemens BioGraph HiRes did not notably alter the results: PD-MCI, 0.79 vs. 0.78 using the whole sample; PDD: 0.88 vs. 0.89 using the whole sample (Supplementary Fig. 8). Additionally, classification models trained directly for the distinction between PD-MCI and PD-CN yielded very similar findings as for the models trained for the distinction between PDD and PD-CN. Classification accuracies in the test set were highest for [^18^F]FDG PET (AUC = 0.81 ± 0.07), followed by dMRI (AUC = 0.73 ± 0.10), and non-significant for sMRI (AUC = 0.62 ± 0.12) (Supplementary Fig. 9A), and classification models relied on similar imaging features (Supplementary Fig. 9B). Finally, classification models discriminating directly between the PD-MCI and PDD groups showed moderate performance for all modalities (AUC: 0.69–0.75; Supplementary Fig. 5B) and there were no statistically significant differences between the modalities for this task (*p* > 0.36).

**Table 2:** Performance metrics for the different classifiers. Different imaging modalities are presented in different rows, and metrics are reported separately for the PD-CN vs. PD-MCI (left) and PD-CN vs. PDD tasks (right)

	AUC	Max(J)	Accuracy	Sensitivity	Specificity
	**PD-CN vs. PD-MCI**				
sMRI	0.57±0.10	0.38±0.13	0.63±0.08	0.73±0.13	0.66±0.13
dMRI	0.71±0.09	0.47±0.12	0.68±0.08	0.76±0.17	0.72±0.14
[^18^F]FDG PET	0.78±0.09	0.53±0.11	0.73±0.07	0.76±0.17	0.77±0.12
	**PD-CN vs. PDD**				
sMRI	0.77±0.07	0.51±0.11	0.69±0.07	0.81±0.15	0.71±0.15
dMRI	0.87±0.06	0.67±0.10	0.77±0.07	0.86±0.13	0.81±0.12
[^18^F]FDG PET	0.89±0.05	0.75±0.10	0.81±0.07	0.89±0.09	0.87±0.10

Abbreviations: AUC, Area under the curve; Max(J), Maximum of the Youden index

### Multi-modality models

**Fig. 3:**
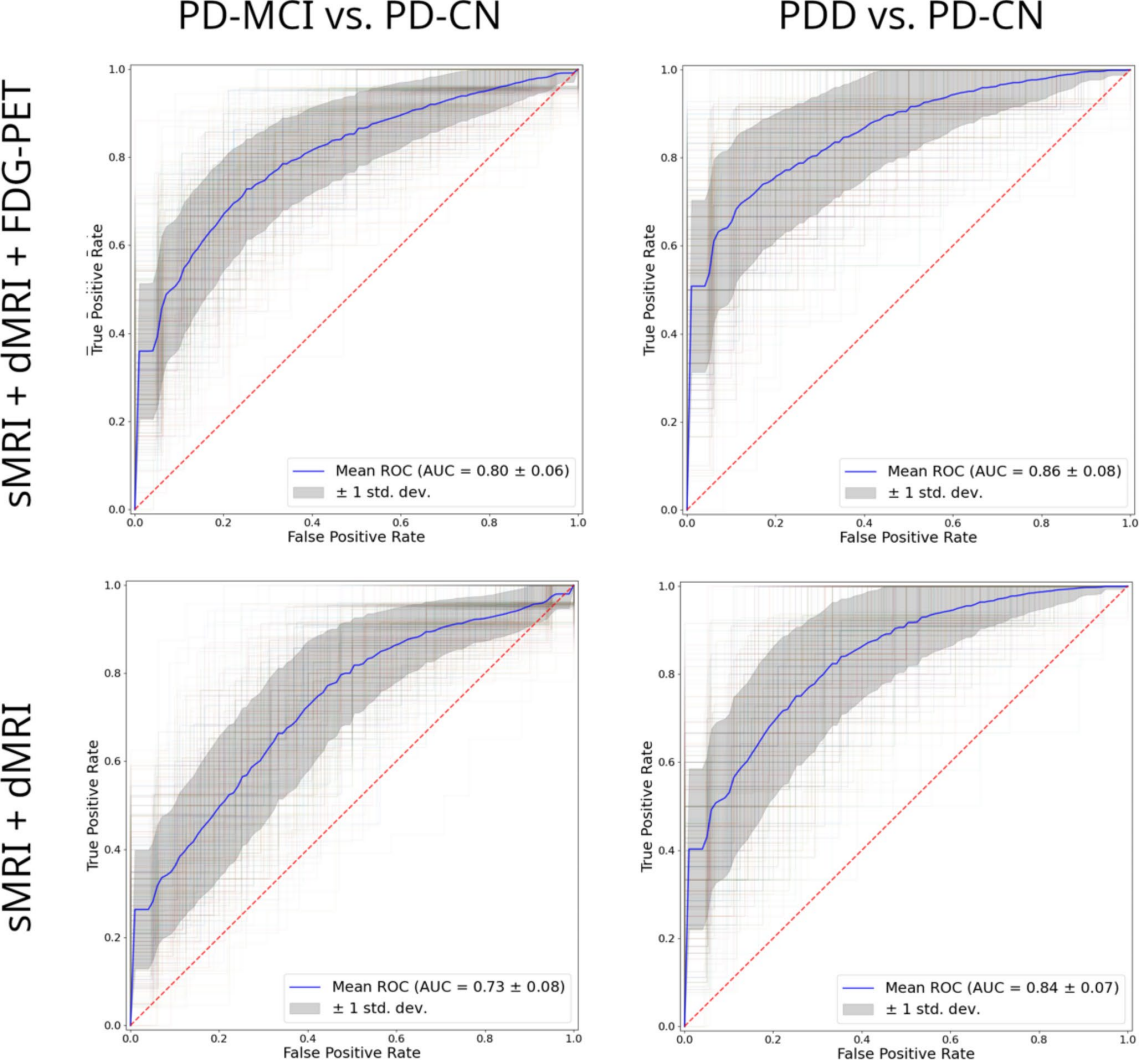
Receiver Operating Characteristic (ROC) curves for the different multi-modality models (blue). Grey areas present the standard deviation of the averaged ROC curves

For the multimodal model including all modalities, the most relevant features were the [^18^F]FDG PET SUVR of the precuneus (26.2% average contribution) and the [^18^F]FDG PET SUVR of the temporooccipital part of the inferior temporal gyrus (25.6%) (Supplementary Fig. 10, top). Notably, of the top ten contributing features, seven came from [^18^F]FDG PET and three came from dMRI, while none came from sMRI. Interestingly, classification models based on the combination of MRI and [^18^F]FDG PET metrics did not show significantly better performance (PDD vs. PD-CN: AUC = 0.86 ± 0.08; PD-MCI vs. PD-CN: AUC of 0.80 ± 0.06; Fig. 3, top) compared to the models based on [^18^F]FDG PET alone (*p* > 0.19). In the model based only on MRI features (Fig. 3, bottom), the features showing the most robust involvement were the hippocampus MD (14.9% average contribution), temporal pole MD (14.8%), parietal operculum cortex MD (10.5%) and inferior frontal gyrus pars opercularis MD (10.5%) (Supplementary Fig. 10, bottom). These MRI-only multimodal models yielded an AUC of 0.84 ± 0.07 for discriminating between PD-CN and PDD and an AUC of 0.73 ± 0.08 for discriminating between PD-CN and PD-MCI. None of these models provided significantly better performance compared to the models based only on dMRI (*p* > 0.57).

## Discussion

While several neuroimaging modalities have shown some promise in detecting the neurodegenerative processes associated with the development of CI in PD, very little work has been done comparing different imaging modalities for this purpose [16]. In addition to providing a better understanding of the relative sensitivities of the available imaging biomarkers, multimodal studies are necessary to understand if different modalities may provide complementary information, thus potentially increasing the accuracy in the evaluation [8]. We used a multimodal imaging approach to directly compare the performance of three of the most widely used neuroimaging modalities (sMRI, dMRI, [^18^F]FDG PET) for detecting cortical changes associated with CI in PD, including both early (PD-MCI) and more advanced (PDD) stages of CI.

Following previous evidence suggesting that affected brain areas may differ between imaging modalities [7, 8, 16], we first conducted exploratory brain-wide analyses aimed at measuring the group-level changes observed within each modality for each stage of the PD cognitive spectrum (PD-MCI, PDD) compared to a group of cognitively unimpaired PD patients. In these analyses, sMRI revealed atrophy in the medial and lateral temporal lobe as well as posterior medial areas (posterior cingulate, precuneus) of PDD patients. This atrophy distribution is similar to that usually observed in patients with Alzheimer’s disease, and has been previously related to CI in PD [10, 34, 35]. Interestingly, however, patients with PD-MCI did not show significant changes on sMRI compared to PD-CN, which aligns with previous research indicating that the pathophysiologic processes underlying early cognitive deficits in PD may not be accurately captured by atrophic changes on sMRI [12, 17, 36, 37]. While this is also in line with previous neuropathological findings suggesting that neuronal loss is a relatively late event in PD pathogenesis [38], some studies have achieved higher accuracies with sMRI using more complex classifiers [9, 39]. Interestingly, dMRI-based diffusion changes in PDD showed a similar topological pattern of abnormalities compared to the atrophic changes on sMRI but were generally more pronounced and widespread. Moreover, dMRI also revealed significant microstructural changes in the medial temporal lobe of PD-MCI patients. These results are in good agreement with previous studies suggesting that cortical microstructural changes measured by dMRI may precede atrophy in the course of the disease [19, 40]. The observed pattern of increased cortical MD was also similar to those found in previous studies [19, 40], suggesting that dMRI may provide more reproducible results than sMRI in the evaluation of PD-CI [7]. Finally, for [^18^F]FDG PET we observed a typical pattern of posterior-occipital hypometabolism in association with CI, which has been extensively described in previous studies [15, 17, 41, 42]. In contrast to the MRI-based modalities, this pattern was already well-defined in PD-MCI, and effect sizes were generally higher compared to both dMRI and sMRI (Fig. 1).

To date, few multimodal imaging studies have conducted direct comparisons between [^18^F]FDG PET and MRI for the assessment of PD-CI, and several authors have highlighted the need for more direct comparisons between different modalities [16, 43]. To the best of our knowledge, our study provides the first direct comparison between dMRI and [^18^F]FDG PET, and very few multimodal studies have conjointly studied [^18^F]FDG PET and sMRI in PD-CI [17]. Our results largely agree with those by González-Redondo et al. [17], who hypothesized that hypometabolism and atrophy represent consecutive stages of the same neurodegeneration process. However, similarly to this previous study, we also observed remarkable topological differences between the hypometabolism and atrophy patterns. Concretely, hypometabolism was most pronounced in posterior-occipital areas, largely sparing the medial temporal lobe, while the opposite was observed for sMRI. These differences were not observed when comparing dMRI and sMRI, which seems to suggest that microstructural changes revealed by dMRI are indeed a closer early prospect of the changes observed in sMRI. Further studies, ideally longitudinal multimodal studies, would be required to provide a better understanding of the relationships between regional patterns of microstructural, macrostructural, and metabolic changes as PD-CI progresses [43].

To further assess the utility of the different neuroimaging biomarkers in a diagnostic context, we trained different classification models aimed at distinguishing PDD and PD-MCI patients from PD-CN patients based on regional image information. For sMRI, the classification models selected GM volumes of medial and lateral temporal regions and the precuneus as the most robust sMRI based features, largely reflecting the temporo-parietal pattern of atrophy in the group-level analysis. However, classifications based on these features provided only chance level discrimination between PD-MCI and PD-CN, indicating low utility of sMRI for this diagnostic context. By contrast, the dMRI classifier, mainly based on MD values of the planum polare, hippocampus, and Heschl’s gyrus, showed a reasonable performance even for PD-MCI (AUC = 0.71). Interestingly, the hippocampus has been previously reported to present diffusivity alterations in dMRI at early stages of PD-related CI, although diagnostic accuracies were not assessed in these previous studies [16, 44, 45]. Finally, [^18^F]FDG PET exhibited an excellent performance for classifying patients with PDD (AUC = 0.89) and even a comparably good performance for PD-MCI (AUC = 0.78). While our study is the first to compare modality-specific diagnostic accuracies in a systematic manner, the superior performance of [^18^F]FDG PET compared to sMRI highlights that the group-level findings from our and other studies [16, 17] translate well to individual patient evaluations. Compared to [^18^F]FDG PET, dMRI performed similarly for classifying PDD patients, but [^18^F]FDG PET outperformed dMRI in the evaluation of PD-MCI patients. This direct multimodal comparison corroborates the conclusion from a recent meta-analysis of uni-modal imaging studies suggesting that [^18^F]FDG PET may outperform dMRI in the evaluation of PD-CI [16]. Future studies may compare [^18^F]FDG PET with other techniques measuring brain function, such as EEG, which has been shown to be very effective in distinguishing PD-CN and PD-MCI [46, 47].

Finally, considering the potential complementary information that can be derived from multiple imaging modalities [31], we assessed additional models combining regional information from several imaging modalities. However, the multimodal models did not statistically outperform the performance of the best-performing uni-modal models, suggesting limited complementary information provided by these multimodal approaches. In summary, we may conclude that multimodal imaging acquisitions do not provide a significant benefit for the evaluation of PD-CI, and researchers and clinicians should use the best performing modality available in their facilities. Based on our analyses, [^18^F]FDG PET may be the most effective imaging modality, with dMRI being a promising MRI-based alternative that clearly outperforms standard volumetric measures in this diagnostic context.

Our study also presents a series of limitations. First, while our study is based on a comparably large sample of well-phenotyped PD patients with multimodal PET-MRI imaging acquisitions [17], it may still be considered relatively small for studying generalizable diagnostic classification models. We have aimed to mitigate data limitations by reducing the complexity of the used modules (linear regression models) and by applying robust cross-validation approaches. Further studies with larger cohorts may also investigate more complex machine learning models (e.g., deep learning) that could potentially further increase diagnostic accuracy [48]. Second, due to the lack of clinical follow-up data we cannot derive any conclusions as to the imaging modalities’ performances for predicting progression to dementia in PD-MCI. While previous uni-modal imaging studies have linked similar imaging abnormalities to a higher risk of imminent clinical progression to dementia in PD [14, 15], this cannot be ascertained in our current cross-sectional study. In this context, we may note that the most sensitive diagnostic imaging features may not necessarily be the ones that provide the highest prognostic value [49], and our classification models distinguishing between PD-MCI and PDD indicate that sMRI may perform better in this context. Longitudinal study designs using multimodal imaging approaches are necessary to address the clinically highly relevant question of whether the different imaging modalities also show differing accuracies in distinguishing between those PD-MCI cases who will or will not progress to dementia. Third, we have limited our analysis to standard metrics of cortical neurodegeneration that are commonly derived from the respective imaging modalities (i.e., SUVR for [^18^F]FDG PET, GM for sMRI, MD for dMRI). Including more comprehensive regional information and/or more advanced imaging metrics could potentially further improve the performance of the tested models, particularly for dMRI [13], which should be explored in further research. Finally, another potential limitation of our study is the absence of a healthy control group, which may hamper the interpretation of the derived neurodegeneration patterns. However, a previous study with a similar study question did include a group of healthy individuals and only found very limited effects of cortical hypometabolism and atrophy in PD-CN subjects as compared to healthy controls [17]. Thus, we may expect that comparisons of PD-MCI and PDD subjects with a healthy control group would reveal very similar cortical neuroimaging patterns as those derived from our comparisons vs. PD-CN.

## Conclusions

[^18^F]FDG PET outperforms dMRI and sMRI for detecting neurodegenerative changes accompanying CI in PD, providing clinically relevant diagnostic information for both PDD and PD-MCI. If limited to MRI acquisitions, dMRI-based diffusion measurements may provide a promising alternative that clearly outperforms standard volumetric measurements derived from sMRI, especially for PD-MCI.

## Electronic supplementary material

Below is the link to the electronic supplementary material.

Supplementary Material 1

## Data Availability

The datasets generated during and/or analyzed during the current study are available from the corresponding author on reasonable request.
