# Supplementary material for: Imaging biomarkers of cortical neurodegeneration underlying cognitive impairment in Parkinson’s disease
Source: Eur J Nucl Med Mol Imaging. 2025 Jan 31;52(6):2002–14. doi: 10.1007/s00259-025-07070-z (PMC12014801; doi:10.1007/s00259-025-07070-z)
Supplement: Supplementary file 1 — Supplementary Material 1 [file 259_2025_7070_MOESM1_ESM.pdf]

Supplementary Figures

Supplementary Figure 1: Cross-validated classification workflow

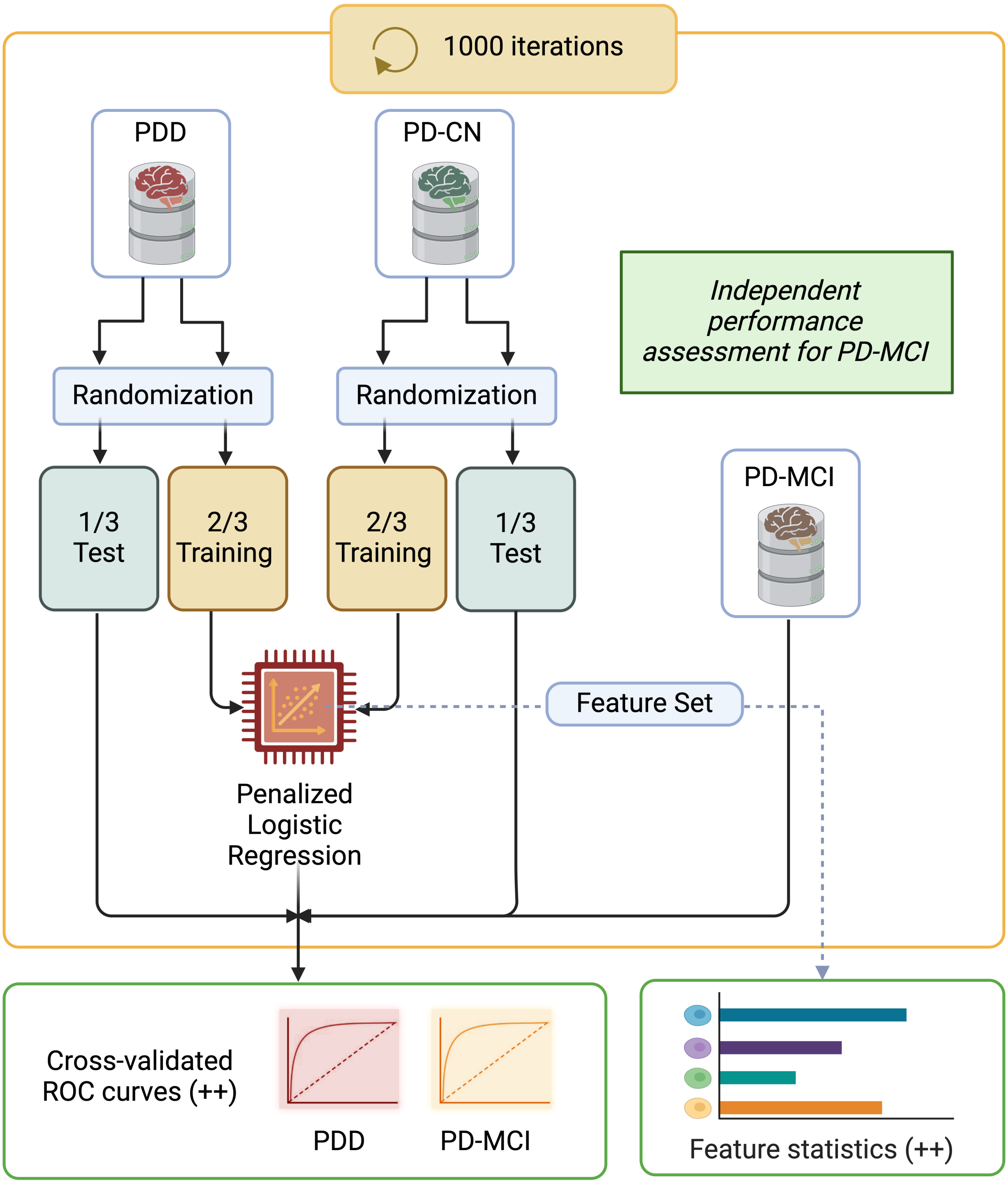

Supplementary Figure 2: ROI analysis results of differences in different diffusion metrics between PD-CN and PD-MCI (left) and between PD-CN and PDD (right): MD (top), FA (middle), and FW (bottom)

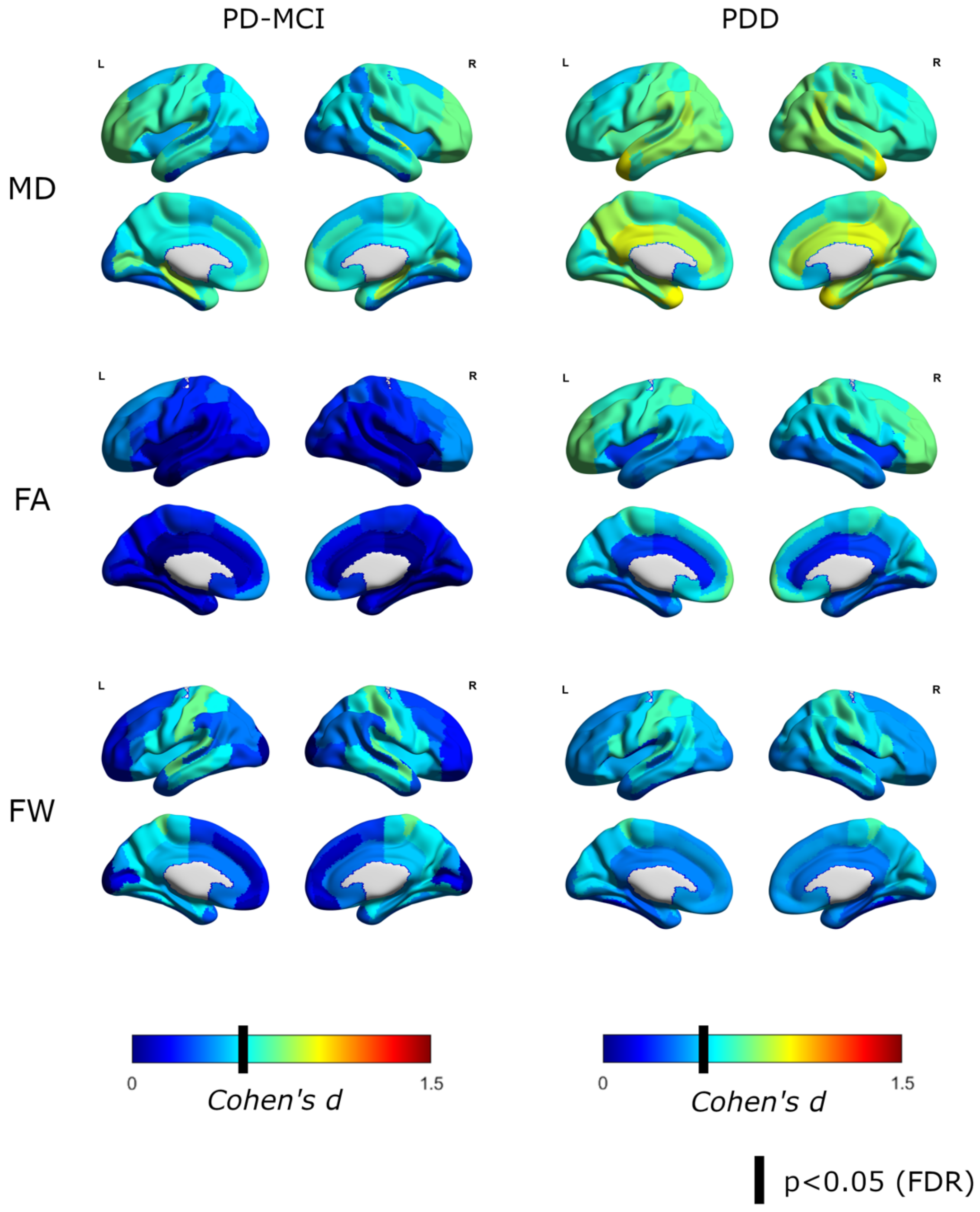

Supplementary Figure 3: ROI analysis results for comparison of PDD vs PD-CN with and without excluding 9 PDD patients that were on cholinergic treatment with rivastigmine.

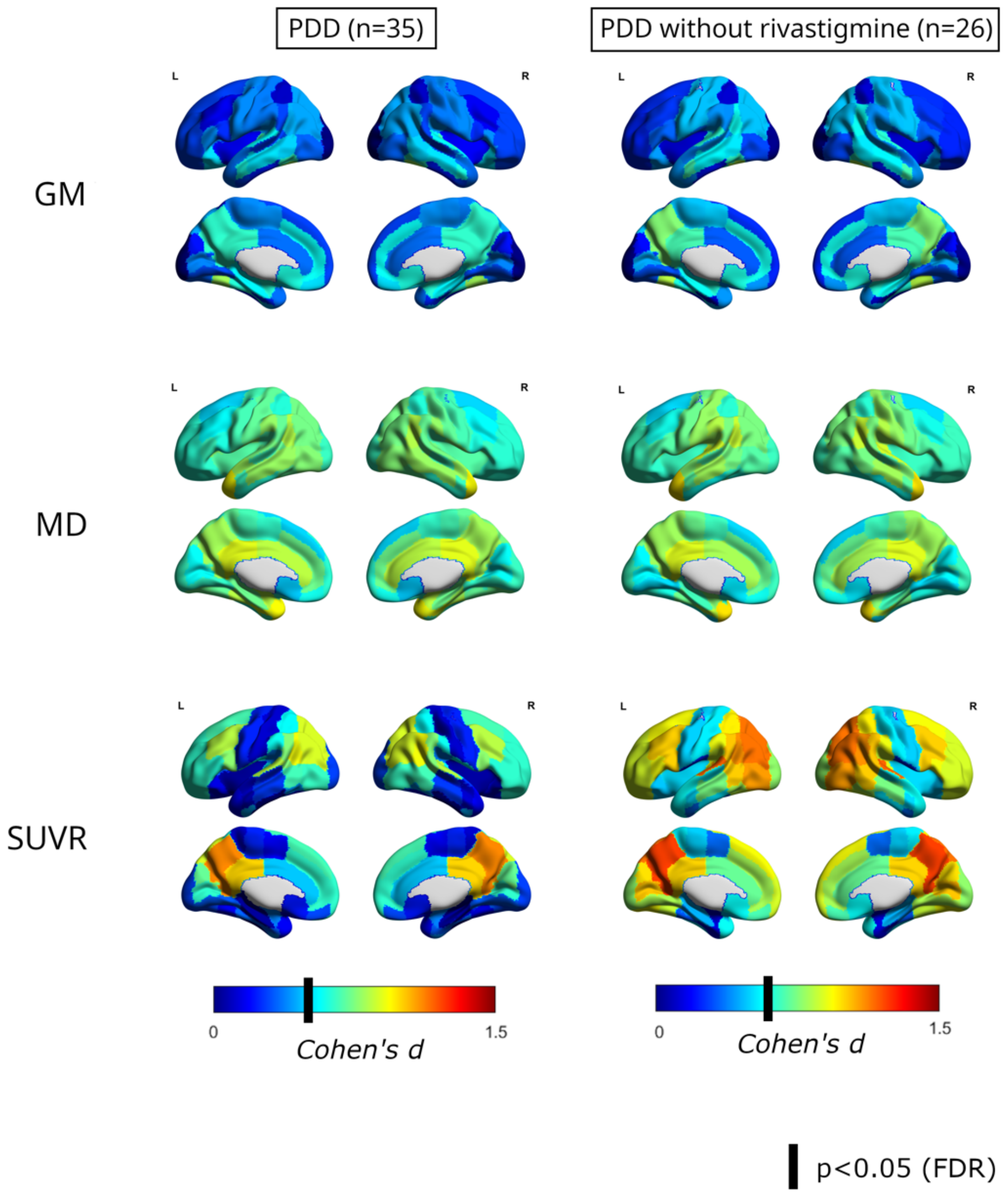

Supplementary Figure 4: ROI analysis results for the comparisons of FDG-PET SUVR values between PD-CN and PD-MCI (left) and PDD (right) with and without excluding 19 patients (9 PD-CN, 5 PD-MCI, 5 PDD) that were acquired using an older scanner.

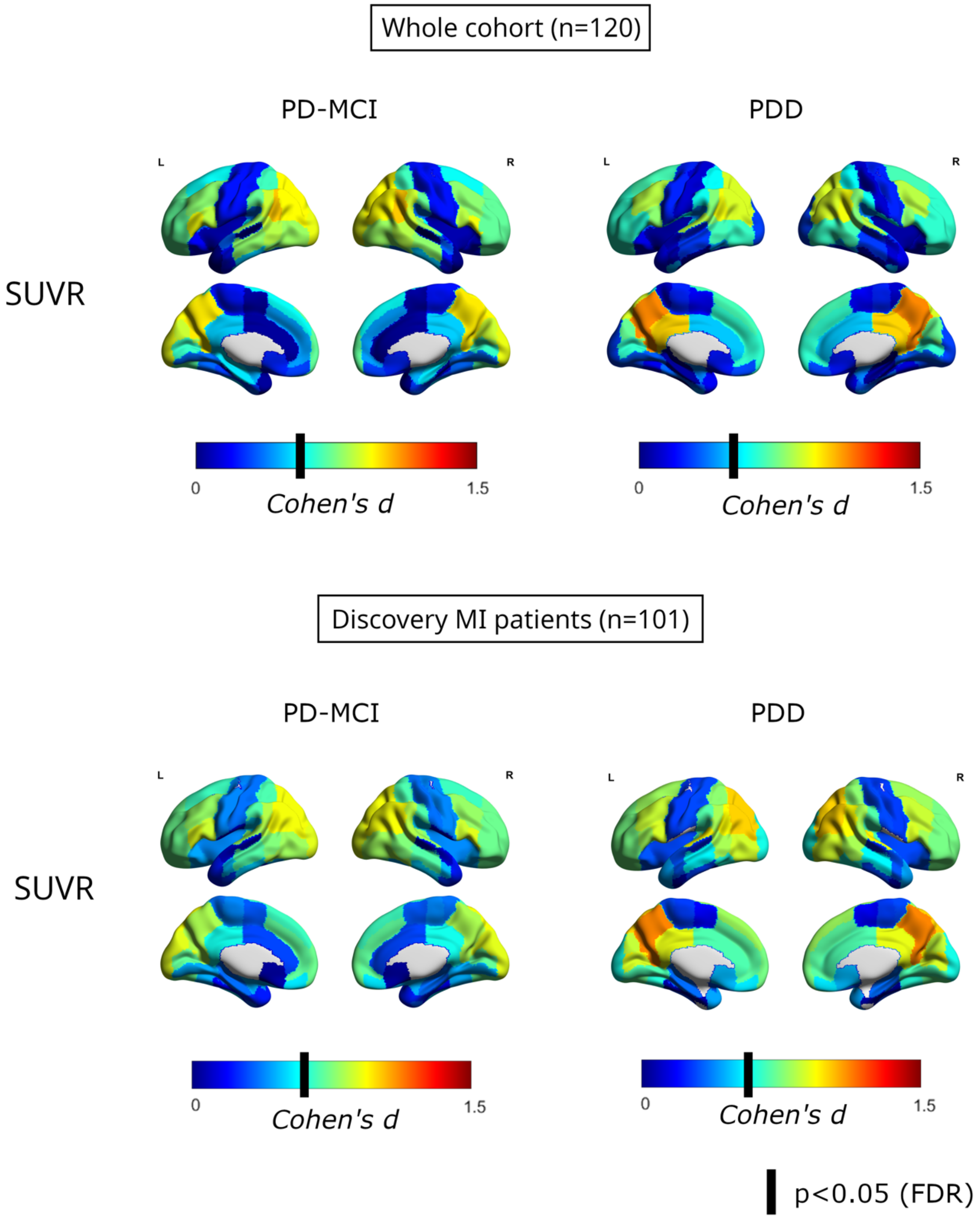

Supplementary Figure 5: (A) ROI analysis results between the PD-MCI and PDD groups. (B) ROC curves of classifiers trained to separate between PD-MCI and PDD.

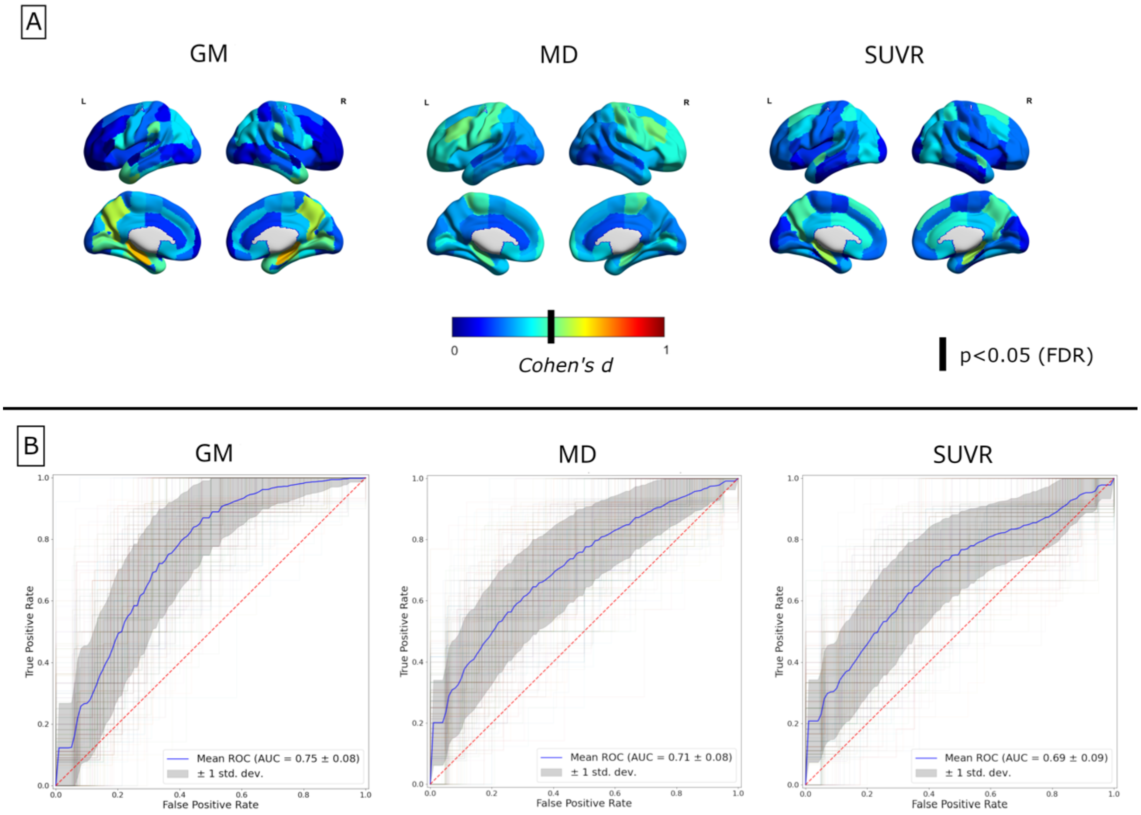

Supplementary Figure 6: Results of the continuous partial Pearson correlation between the PD-CRS and ROI values of the three modalities (top). The independent effects of UPDRS-III and age, which were used as covariates for the main analysis, are also displayed.

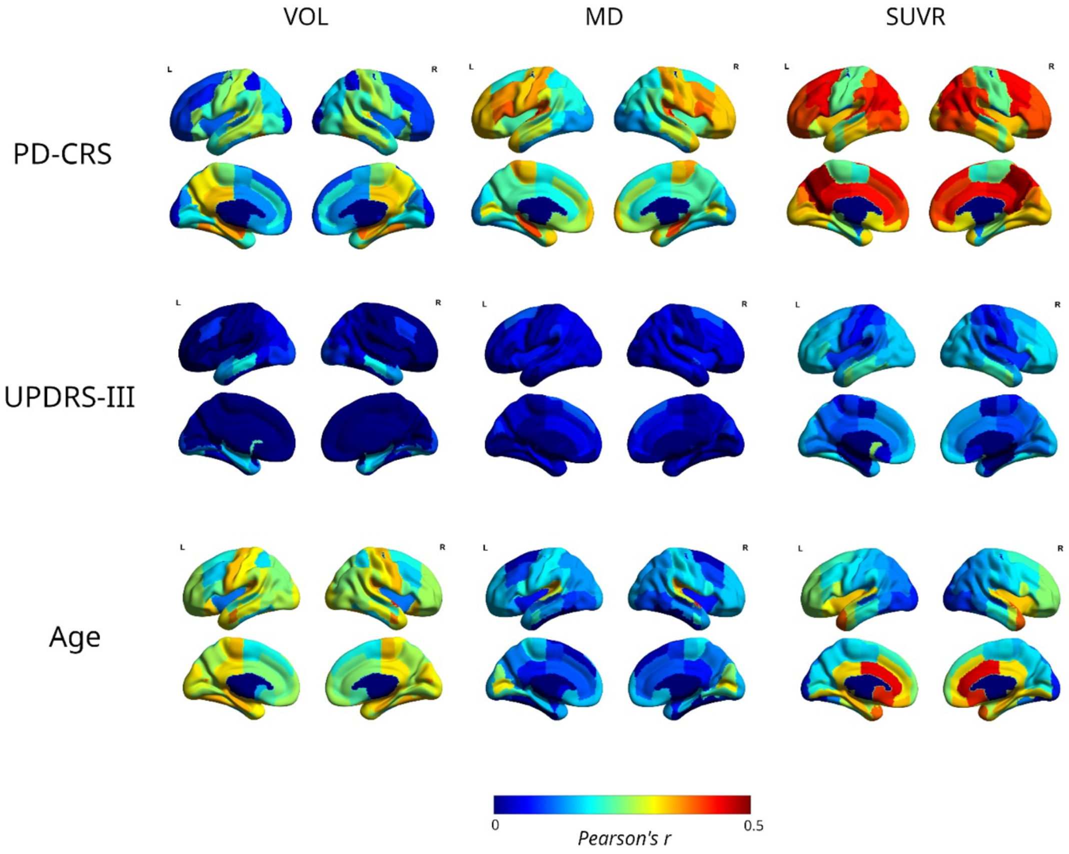

Supplementary Figure 7: Relative contributions of the top ten contributing ROIs for each of the modality-specific models.

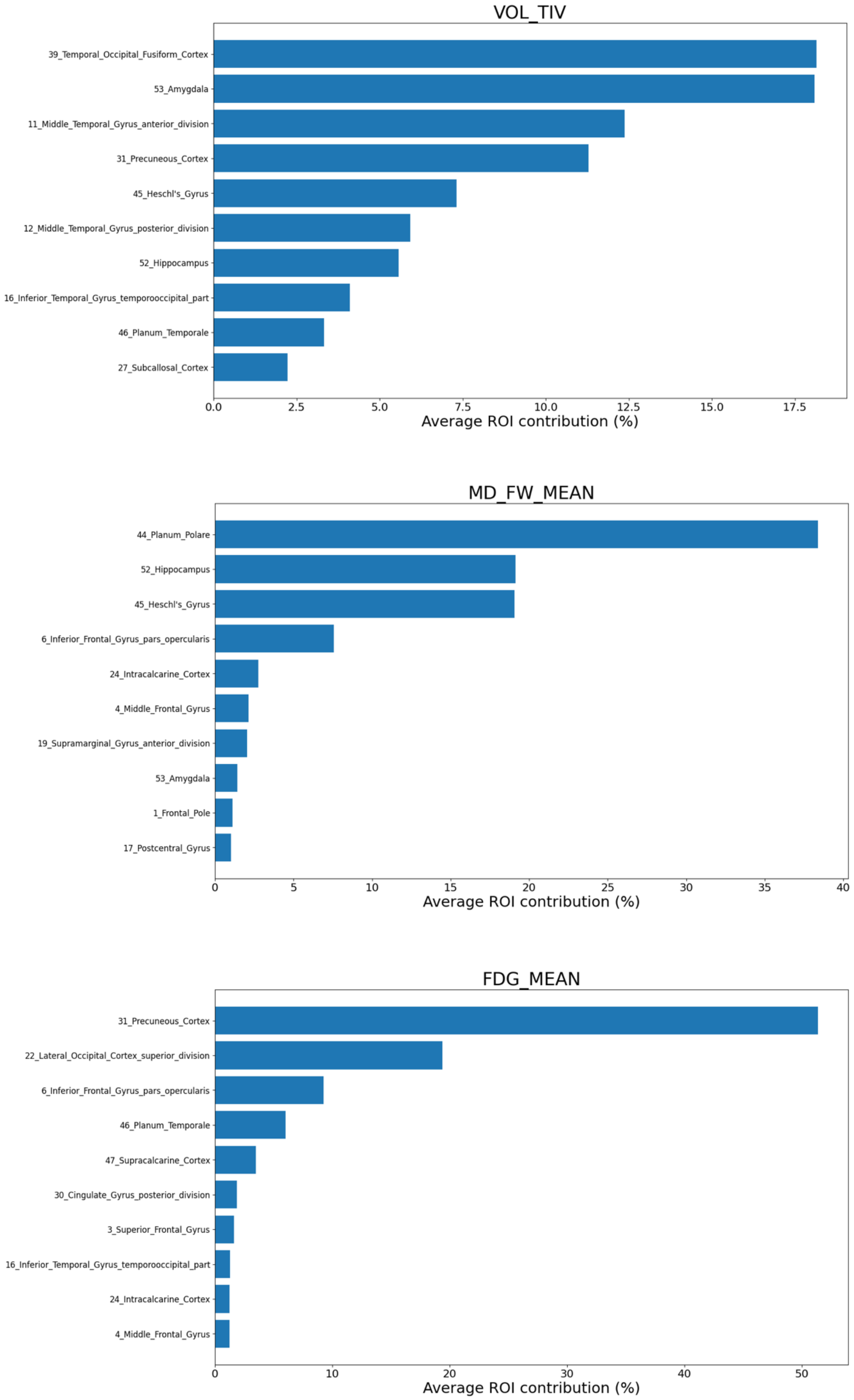

Supplementary Figure 8: Classification results for FDG-PET with and without excluding 19 patients (9 PD-CN, 5 PD-MCI, 5 PDD) that were acquired using an older scanner.

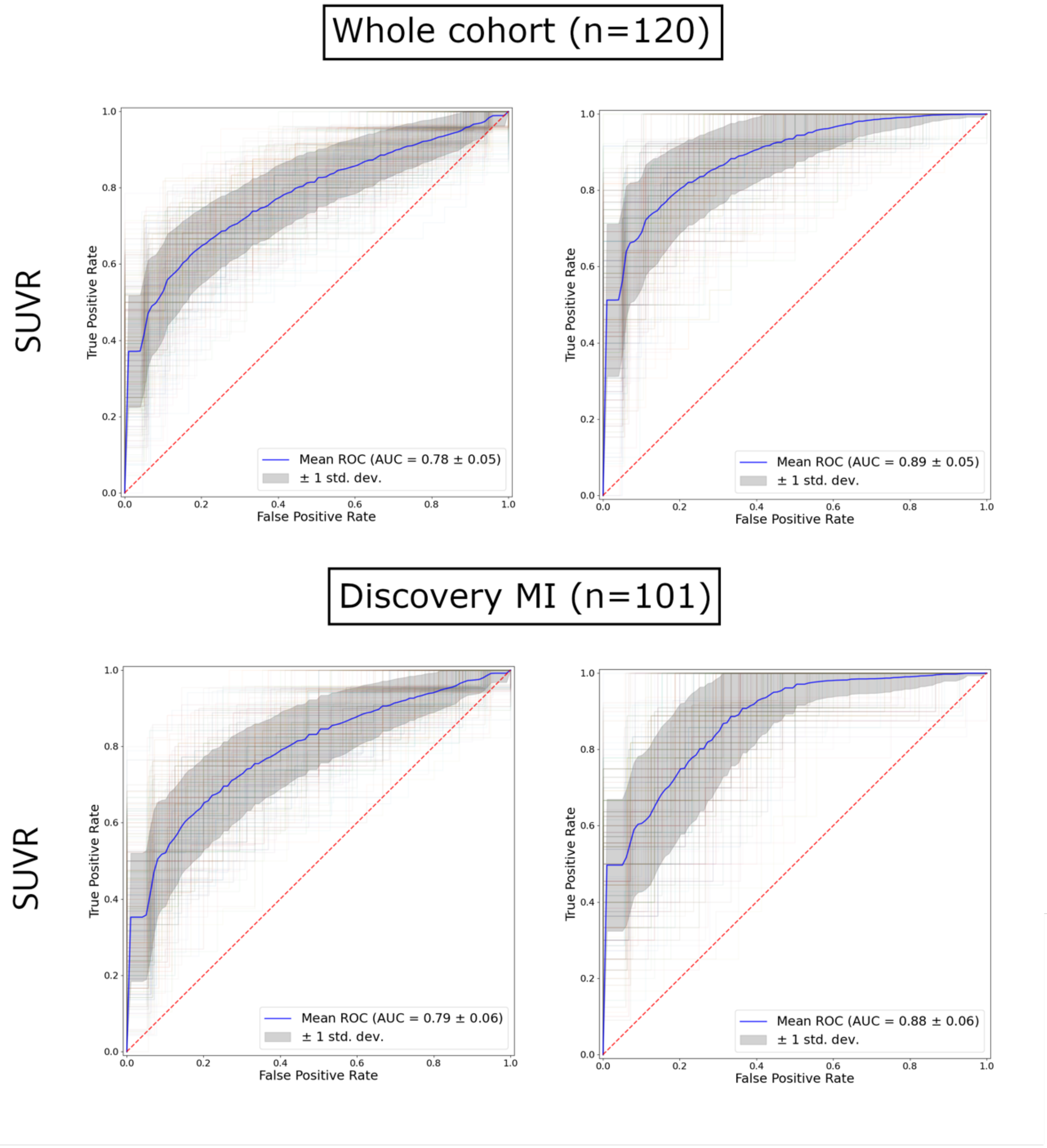

Supplementary Figure 9: A) Receiver Operating Characteristic (ROC) curves for the different modality-specific models trained directly for the distinction between PD-MCI and PD-CN (blue lines). Grey areas present the standard deviation of the averaged ROC curves. B) Relative contributions of the top ten contributing ROIs for each of the models.

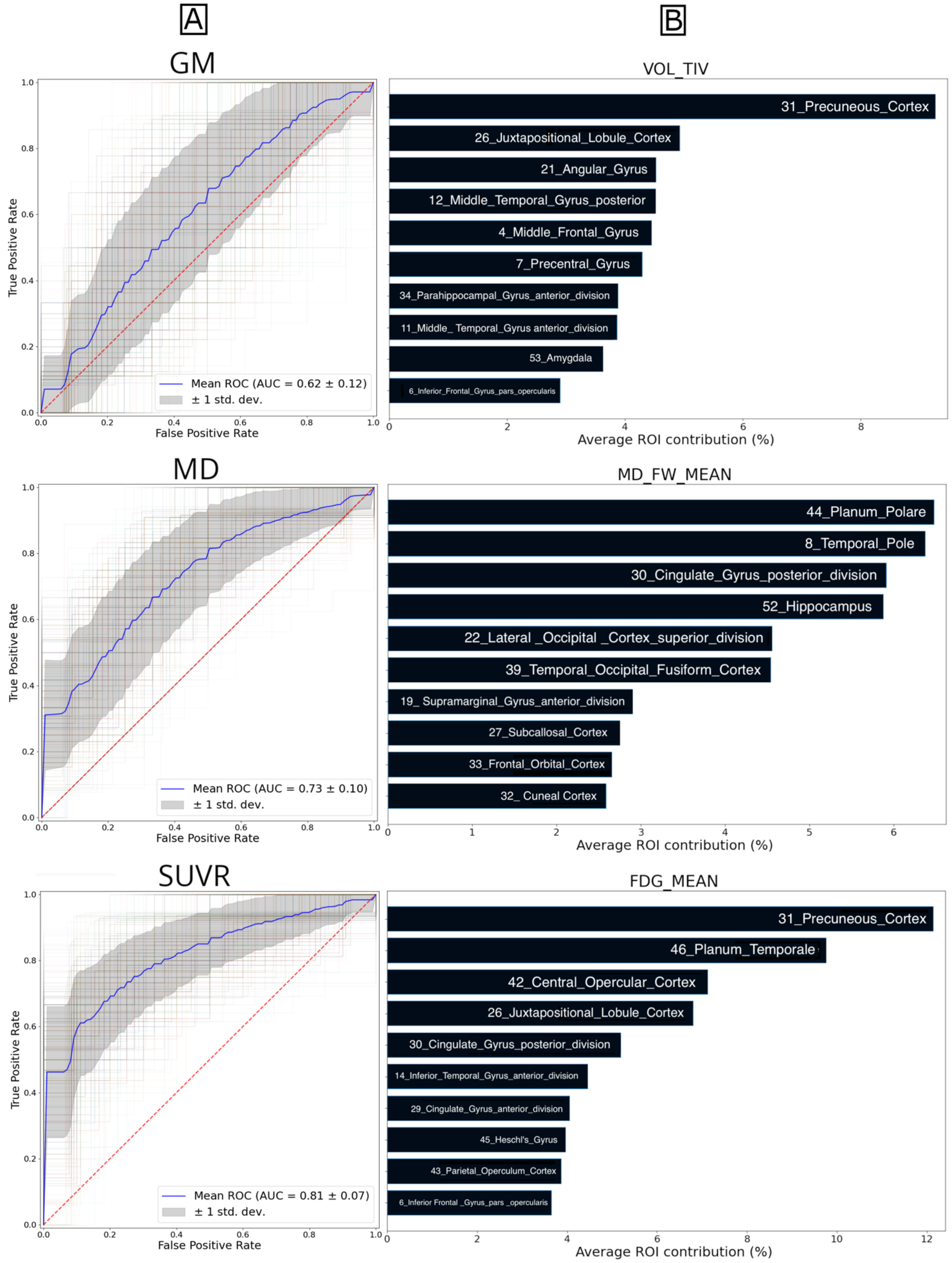

Supplementary Figure 10: Relative contributions of ROI measurements from different modalities in the multimodal models.

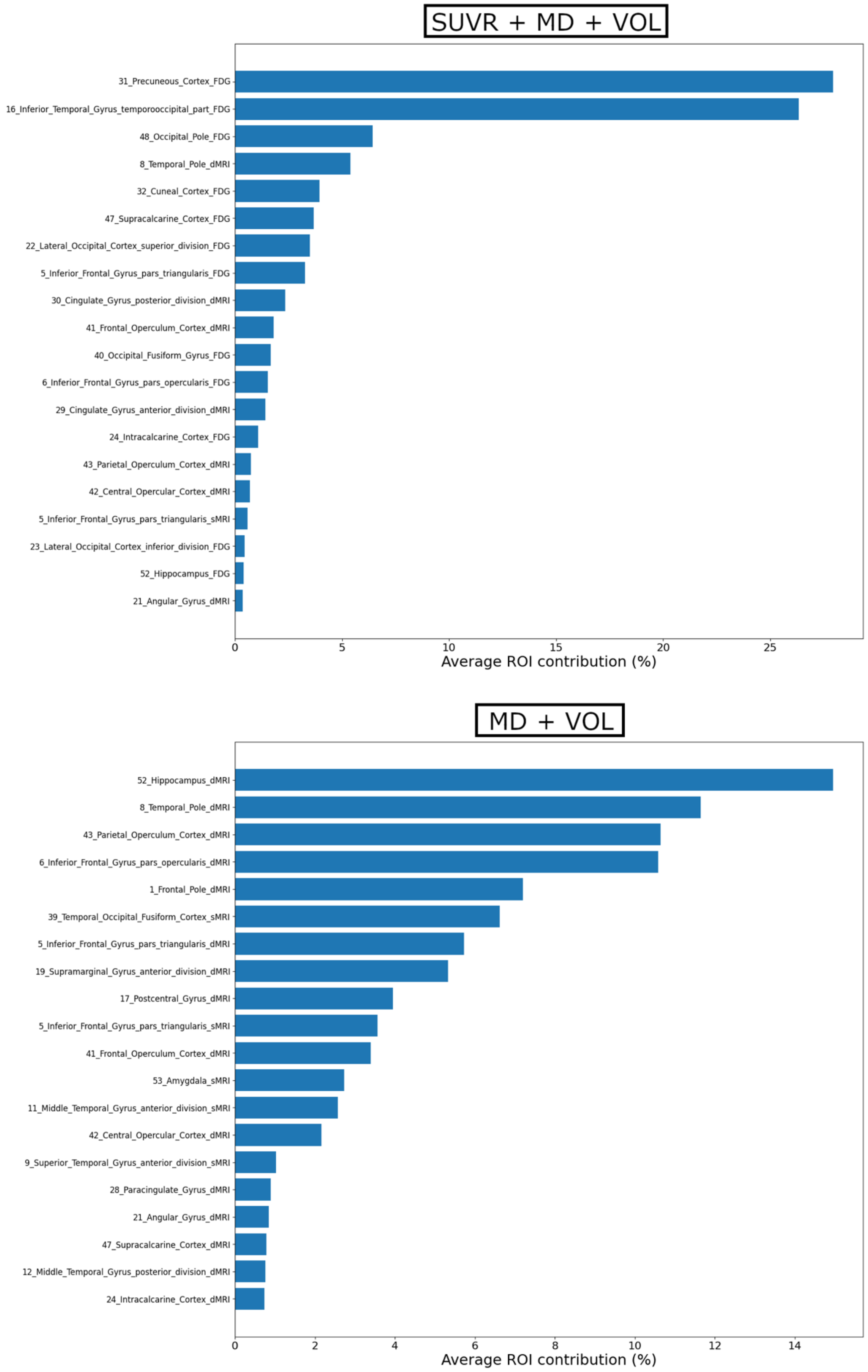

Supplementary Tables

Supplementary Table 1. Cohen's d values and FDR-corrected p-values of GM_Vol ROI-based comparisons. Models are corrected for age and UPDRS-III.

	PD-MCI		PDD	
**Harvard Oxford ROI**	**Cohens d**	p (FDR-corr)	**Cohens d**	p (FDR-corr)
1 Frontal Pole	0,134	0,563	0,289	0,229
2 Insular Cortex	0,192	0,407	0,182	0,422
3 Superior Frontal Gyrus	0,332	0,153	0,286	0,229
4 Middle Frontal Gyrus	0,107	0,642	0,177	0,427
5 Inferior Frontal Gyrus pars triangularis	0,304	0,191	0,401	0,133
6 Inferior Frontal Gyrus pars opercularis	0,209	0,366	0,255	0,274
7 Precentral Gyrus	0,056	0,808	0,395	0,136
8 Temporal Pole	0,167	0,470	0,354	0,155
9 Superior Temporal Gyrus anterior division	0,245	0,291	0,367	0,148
10 Superior Temporal Gyrus posterior division	0,164	0,478	0,269	0,251
11 Middle Temporal Gyrus anterior division	0,019	0,934	0,600	0,033
12 Middle Temporal Gyrus posterior division	0,084	0,716	0,528	0,050
13 Middle Temporal Gyrus temporooccipital part	0,164	0,479	0,504	0,056
14 Inferior Temporal Gyrus anterior division	0,221	0,341	0,282	0,232
15 Inferior Temporal Gyrus posterior division	0,350	0,133	0,222	0,334
16 Inferior Temporal Gyrus temporooccipital part	0,174	0,453	0,643	0,033
17 Postcentral Gyrus	0,298	0,199	0,374	0,148
18 Superior Parietal Lobule	0,102	0,659	0,105	0,636
19 Supramarginal Gyrus anterior division	0,053	0,819	0,369	0,148
20 Supramarginal Gyrus posterior division	0,010	0,965	0,290	0,229
21 Angular Gyrus	0,177	0,445	0,450	0,095
22 Lateral Occipital Cortex superior division	0,125	0,588	0,436	0,105
23 Lateral Occipital Cortex inferior division	0,037	0,874	0,337	0,169
24 Intracalcarine Cortex	0,389	0,096	0,387	0,140
25 Frontal Medial Cortex	0,199	0,390	0,520	0,050
26 Juxtapositional Lobule Cortex	0,124	0,591	0,406	0,130
27 Subcallosal Cortex	0,107	0,643	0,607	0,033
28 Paracingulate Gyrus	0,072	0,757	0,576	0,040
29 Cingulate Gyrus anterior division	0,107	0,643	0,353	0,155
30 Cingulate Gyrus posterior division	0,065	0,780	0,636	0,033
31 Precuneous Cortex	0,138	0,551	0,632	0,033
32 Cuneal Cortex	0,162	0,484	0,212	0,350
33 Frontal Orbital Cortex	0,042	0,855	0,525	0,050
34 Parahippocampal Gyrus anterior division	0,281	0,226	0,241	0,298
35 Parahippocampal Gyrus posterior division	0,118	0,611	0,532	0,050
36 Lingual Gyrus	0,226	0,329	0,301	0,225
37 Temporal Fusiform Cortex anterior division	0,017	0,941	0,454	0,095
38 Temporal Fusiform Cortex posterior division	0,047	0,839	0,620	0,033
39 Temporal Occipital Fusiform Cortex	0,135	0,559	0,805	0,033
40 Occipital Fusiform Gyrus	0,069	0,766	0,337	0,169
41 Frontal Operculum Cortex	0,003	0,988	0,367	0,148
42 Central Opercular Cortex	0,230	0,322	0,354	0,155
43 Parietal Operculum Cortex	0,046	0,843	0,412	0,130
44 Planum Polare	0,052	0,823	0,543	0,050
45 Heschl's Gyrus	0,234	0,312	0,667	0,033
46 Planum Temporale	0,373	0,109	0,567	0,041
47 Supracalcarine Cortex	0,057	0,806	0,289	0,229
48 Occipital Pole	0,346	0,137	0,005	0,983
52 Hippocampus	0,259	0,265	0,554	0,046
53 Amygdala	0,044	0,850	0,619	0,033

Supplementary Table 2. Cohen's d values and FDR-corrected p-values of MD ROI-based comparisons. Models are corrected for age and UPDRS-III.

	**PD-MCI**		**PDD**	
**Harvard Oxford ROI**	**Cohens d**	p (FDR-corr)	**Cohens d**	p (FDR-corr)
1 Frontal Pole	0,729	0,0091	0,627	0,010
2 Insular Cortex	0,431	0,0598	0,689	0,007
3 Superior Frontal Gyrus	0,486	0,0379	0,508	0,031
4 Middle Frontal Gyrus	0,659	0,0107	0,567	0,017
5 Inferior Frontal Gyrus pars triangularis	0,700	0,0091	0,650	0,008
6 Inferior Frontal Gyrus pars opercularis	0,671	0,0091	0,607	0,011
7 Precentral Gyrus	0,618	0,0132	0,666	0,008
8 Temporal Pole	0,671	0,0091	0,931	<0,001
9 Superior Temporal Gyrus anterior division	0,530	0,0242	0,798	0,003
10 Superior Temporal Gyrus posterior division	0,441	0,0583	0,657	0,008
11 Middle Temporal Gyrus anterior division	0,624	0,0111	0,651	0,008
12 Middle Temporal Gyrus posterior division	0,635	0,0111	0,789	0,003
13 Middle Temporal Gyrus temporooccipital part	0,433	0,0598	0,791	0,003
14 Inferior Temporal Gyrus anterior division	0,262	0,2230	0,621	0,010
15 Inferior Temporal Gyrus posterior division	0,427	0,0598	0,622	0,010
16 Inferior Temporal Gyrus temporooccipital part	0,336	0,1277	0,709	0,006
17 Postcentral Gyrus	0,647	0,0107	0,709	0,006
18 Superior Parietal Lobule	0,380	0,0919	0,616	0,010
19 Supramarginal Gyrus anterior division	0,585	0,0146	0,784	0,003
20 Supramarginal Gyrus posterior division	0,428	0,0598	0,715	0,006
21 Angular Gyrus	0,563	0,0185	0,826	0,003
22 Lateral Occipital Cortex superior division	0,558	0,0196	0,737	0,005
23 Lateral Occipital Cortex inferior division	0,375	0,0932	0,759	0,005
24 Intracalcarine Cortex	0,723	0,0091	0,572	0,016
25 Frontal Medial Cortex	0,690	0,0091	0,590	0,013
26 Juxtapositional Lobule Cortex	0,470	0,0429	0,636	0,009
27 Subcallosal Cortex	0,586	0,0146	0,489	0,037
28 Paracingulate Gyrus	0,641	0,0111	0,727	0,005
29 Cingulate Gyrus anterior division	0,518	0,0266	0,838	0,003
30 Cingulate Gyrus posterior division	0,585	0,0146	0,898	<0,001
31 Precuneous Cortex	0,597	0,0146	0,805	0,003
32 Cuneal Cortex	0,531	0,0242	0,570	0,017
33 Frontal Orbital Cortex	0,581	0,0160	0,632	0,010
34 Parahippocampal Gyrus anterior division	0,431	0,0598	0,910	<0,001
35 Parahippocampal Gyrus posterior division	0,572	0,0173	0,800	0,003
36 Lingual Gyrus	0,479	0,0412	0,638	0,009
37 Temporal Fusiform Cortex anterior division	0,317	0,1439	0,662	0,008
38 Temporal Fusiform Cortex posterior division	0,379	0,0919	0,835	0,003
39 Temporal Occipital Fusiform Cortex	0,323	0,1396	0,711	0,006
40 Occipital Fusiform Gyrus	0,339	0,1261	0,725	0,005
41 Frontal Operculum Cortex	0,688	0,0091	0,768	0,003
42 Central Opercular Cortex	0,649	0,0107	0,754	0,005
43 Parietal Operculum Cortex	0,675	0,0091	0,673	0,008
44 Planum Polare	0,889	<0,001	0,791	0,003
45 Heschl's Gyrus	0,766	0,0091	0,715	0,006
46 Planum Temporale	0,626	0,0111	0,742	0,005
47 Supracalcarine Cortex	0,541	0,0224	0,653	0,008
48 Occipital Pole	0,348	0,1189	0,648	0,008
52 Hippocampus	0,829	<0,001	0,726	0,005
53 Amygdala	0,593	0,0146	0,837	0,003

Supplementary Table 3. Cohen's d values and FDR-corrected p-values of SUVR ROI-based comparisons. Models are corrected for age and UPDRS-III.

	PD-MCI		PDD	
**Harvard Oxford ROI**	**Cohens d**	p (FDR-corr)	**Cohens d**	p (FDR-corr)
1 Frontal Pole	0,709	0,008	0,634	0,013
2 Insular Cortex	0,071	0,825	0,052	0,842
3 Superior Frontal Gyrus	0,574	0,036	0,670	0,009
4 Middle Frontal Gyrus	0,768	0,003	0,835	<0,001
5 Inferior Frontal Gyrus pars triangularis	0,922	<0,001	0,618	0,015
6 Inferior Frontal Gyrus pars opercularis	0,833	0,003	0,770	0,005
7 Precentral Gyrus	0,216	0,462	0,239	0,359
8 Temporal Pole	0,043	0,870	0,178	0,496
9 Superior Temporal Gyrus anterior division	0,331	0,242	0,115	0,641
10 Superior Temporal Gyrus posterior division	0,034	0,882	0,234	0,363
11 Middle Temporal Gyrus anterior division	0,205	0,463	0,125	0,638
12 Middle Temporal Gyrus posterior division	0,775	0,003	0,275	0,286
13 Middle Temporal Gyrus temporooccipital part	0,829	0,003	0,664	0,012
14 Inferior Temporal Gyrus anterior division	0,192	0,485	0,441	0,091
15 Inferior Temporal Gyrus posterior division	0,477	0,082	0,006	0,977
16 Inferior Temporal Gyrus temporooccipital part	0,769	0,003	0,418	0,110
17 Postcentral Gyrus	0,212	0,462	0,119	0,641
18 Superior Parietal Lobule	0,657	0,013	0,569	0,024
19 Supramarginal Gyrus anterior division	0,716	0,008	0,583	0,022
20 Supramarginal Gyrus posterior division	0,863	<0,001	0,756	0,005
21 Angular Gyrus	1,017	<0,001	0,920	<0,001
22 Lateral Occipital Cortex superior division	0,937	<0,001	0,882	<0,001
23 Lateral Occipital Cortex inferior division	0,790	0,003	0,647	0,012
24 Intracalcarine Cortex	0,645	0,015	0,636	0,013
25 Frontal Medial Cortex	0,282	0,320	0,295	0,266
26 Juxtapositional Lobule Cortex	0,051	0,870	0,179	0,496
27 Subcallosal Cortex	0,239	0,408	0,219	0,395
28 Paracingulate Gyrus	0,420	0,133	0,634	0,013
29 Cingulate Gyrus anterior division	0,043	0,870	0,482	0,059
30 Cingulate Gyrus posterior division	0,492	0,075	0,996	<0,001
31 Precuneous Cortex	0,965	<0,001	1,110	<0,001
32 Cuneal Cortex	0,860	<0,001	0,715	0,005
33 Frontal Orbital Cortex	0,179	0,505	0,151	0,559
34 Parahippocampal Gyrus anterior division	0,272	0,336	0,317	0,227
35 Parahippocampal Gyrus posterior division	0,100	0,739	0,099	0,685
36 Lingual Gyrus	0,347	0,227	0,322	0,225
37 Temporal Fusiform Cortex anterior division	0,322	0,252	0,406	0,120
38 Temporal Fusiform Cortex posterior division	0,285	0,320	0,340	0,198
39 Temporal Occipital Fusiform Cortex	0,341	0,229	0,163	0,532
40 Occipital Fusiform Gyrus	0,695	0,008	0,290	0,268
41 Frontal Operculum Cortex	0,364	0,211	0,345	0,195
42 Central Opercular Cortex	0,177	0,505	0,563	0,025
43 Parietal Operculum Cortex	0,356	0,217	0,380	0,144
44 Planum Polare	0,203	0,463	0,269	0,294
45 Heschl's Gyrus	0,561	0,039	0,557	0,026
46 Planum Temporale	0,790	0,003	0,836	<0,001
47 Supracalcarine Cortex	0,798	0,003	0,744	0,005
48 Occipital Pole	0,906	<0,001	0,285	0,272
52 Hippocampus	0,530	0,052	0,045	0,849
53 Amygdala	0,448	0,106	0,382	0,144
